# Predictors of coronary artery calcification and its association with cardiovascular events in patients with chronic kidney disease

**DOI:** 10.1080/0886022X.2021.1953529

**Published:** 2021-07-27

**Authors:** Xue-rong Wang, Liang- Yuan, Rui- Shi, Huan- Li, De-guang Wang, Yong-gui Wu

**Affiliations:** a Department of Nephrology, The Second Hospital of Anhui Medical University, Hefei, PR China; b Department of Radiology, The Second Hospital of Anhui Medical University, Hefei, PR China; c Department of Nephrology, The First Affiliated Hospital of Anhui Medical University, Hefei, PR China

**Keywords:** Chronic kidney disease, coronary artery calcification, cardiovascular events, matrix Gla protein, neutrophil-lymphocyte ratio

## Abstract

**Objective:**

To investigate the predictors of coronary artery calcification (CAC) and its association with cardiovascular events (CVE) in patients with stage 3–5 chronic kidney disease (CKD).

**Method:**

Two hundred ninety CKD patients in our nephrology department were enrolled from 2018 to May 2019. The levels of matrix Gla protein (MGP) and interleukin 6 (IL-6) were measured *via* enzyme-linked immunosorbent assay (ELISA) method in 131 CKD patients of all. CAC was evaluated *via* computed tomography (CT). The covariate factors were analyzed by binary logistic regression analysis. We conducted the visits to explore the prevalence of CVE in 276 CKD patients, and covariate factors were analyzed by Cox proportional hazard model.

**Results:**

The prevalence of CAC was up to 57.93%. We found that age, diabetes mellitus, hyperphosphatemia, dialysis duration, and the neutrophil-lymphocyte ratio (NLR) were positively associated with CAC in all patients. In 131 patients, we demonstrated that higher IL-6 and lower MGP levels were associated with CAC. A Cox proportional hazard model demonstrated that moderate to severe CAC was correlated with an increased risk for CVE [Hazard Ratio (HR): 7.250; 95% confidence interval (CI): 3.192–16.470], and a higher MGP level was associated with a reduced risk for CVE (HR: 0.340; 95% CI: 0.124–0.933).

**Conclusions:**

The prevalence of CAC in patients with CKD is a significant issue. Older age, hyperphosphatemia, dialysis duration, diabetes mellitus, IL-6, and the NLR are associated with CAC. In addition, higher MGP levels represent protective factor for CAC. Moderate to severe CAC, and lower MGP levels are associated with an increased risk for CVE. **Abbreviations**: AGEs: Advanced glycosylation end products; CAC: Coronary artery calcification; CACS: Coronary artery calcification score; Ca: Calcium; CI: confidence interval; CKD: Chronic kidney disease; CVE: Cardiovascular events; CT: Computed tomography; ELISA: Enzyme-linked immunosorbent assay; Hb: hemoglobin; HR: Hazard ratio; hs-CRP: high-sensitivity C-reactive protein; IL-6: Interleukin 6; iPTH: Intact parathyroid hormone; Mg: Magnesium; MGP: Matrix Gla protein; NF-κB: nuclear factor-kappa gene binding; NRL: Neutrophil-lymphocyte ratio; Runx2: Runt-related transcription factor 2; RRT: Renal replacement therapy; P: Phosphorus; Scr: Serum creatinine; TNF--alpha: Tumor necrosis factor--alpha; TC: Total cholesterol; TG: Triglyceride; VSMC: vascular smooth muscle cel

## Introduction

Chronic kidney disease (CKD) is highly prevalent and represents a serious threat to public health. The prevalence of CKD is estimated at approximately 10.8%, with nearly 1.2 million persons being affected by this disorder in China [1]. Russo et al. have reported that coronary artery calcification (CAC) was more prevalent in patients with diabetes mellitus [2]. Additionally, the risk for death in patients with CKD has been estimated to be 8 times higher than that in the general population, and CVD accounts for more than 50% of the deaths in patients with CKD [3]. Vascular calcification is an important risk factor for CVD in patients with CKD, and approximately 54–100% of patients have vascular calcifications [4,5].

Vascular calcification is divided into two classifications: intimal calcification and medial calcification. Arterial media calcification is more common in CKD patients. Moreover, intimal calcification of coronary artery is often accompanied by atherosclerosis, and severe cases result in myocardial ischemia and infarction. If CAC is located in the medial membrane according to different populations and modalities of dialysis, then it can deteriorate vascular compliance, thus resulting in left ventricular hypertrophy and heart failure. Once CAC appears, it is difficult to halt the progression. Therefore, it is very important to explore the risk factors for vascular calcification and to prevent a worsened progression over time.

Micro-inflammation is more common in CKD patients. The existence of micro-inflammation is related to a variety of reasons, such as malnutrition, oxidative stress, and endotoxin contamination in dialysate. IL-6 and tumor necrosis factor-alpha (TNF-alpha) are common indicators of micro-inflammatory status. Several studies reported that the level of IL-6 was higher in patients with vascular calcification, other studies demonstrated that serum TNF-alpha concentration was not associated with vascular calcification [6,7]. Therefore, it is necessary to explore whether the microinflammatory state is closely related to vascular calcification. Additionally, the neutrophil-lymphocyte ratio (NLR) is positively correlated with atherosclerosis, acute coronary syndrome, and cancer [8,9]. However, the NLR has not been elucidated in patients with CAC. The matrix protein Gla acts as a protective factor for vascular calcification in animal experiments, and few studies have been performed in patients with CKD [10,11]^.^

Therefore, we performed this study to explore the factors of CAC and its association with CVEs.

## Materials and methods

### Study samples

Two hundred ninety CKD patients were enrolled in our study, and this study was prospective in nature. All of the patients completed CAC Agatston scores. Additionally, serum was collected from 131 CKD patients obtained in our centers (the Department of Nephrology at the Second Hospital of Anhui Medical University). Healthy control (*n* = 45) samples were selected from the Center of Health Examination in our hospital. This study was approved by the ethics committee for human research at the Second Hospital of Anhui Medical University (NO:PJ-YX2017-013), and all of the participants provided written informed prior to their entry into the study.

The following inclusion criteria were used: (1) patients older than 18-year-old; (2) patients who were at stage 3–5 CKD; (3) CAC examinations were performed in our hospital, and the Agatston score was used to evaluate the degree of CAC. The following exclusion criteria were used: (1) patients who had undergone renal replacement therapy for fewer than three months; (2) patients with incidences of acute inflammation in the last month, with acute inflammation being defined as respiratory or urinary tract infections, gastrointestinal infections, or catheter-related infections; (3) patients who had severe cardiovascular diseases, such as congestive heart disease, myocardial infarctions, and coronary artery disease; (4) patients with advanced tumors.

### Biochemical measurements

Fasting blood samples were obtained for determinations of albumin, cholesterol, triglyceride, creatinine, 25-Hydroxy vitamin D, alkaline phosphatase, high-sensitivity C-reactive protein, intact parathyroid hormone (iPTH), calcium, and phosphorus levels. The serum was isolated and stored at −80 °C before use. The IL-6 level was measured by a human ELISA kit (Elabscience, Wuhan, China). Serum MGP was assayed by the use of a human ELISA kit (Cloud Clone Corp., Beijing, China).

### Coronary artery calcification assessment

Coronary artery calcification was quantified using non-contrast chest CT in our hospital according to special criteria, the Agatston score was used to evaluate CAC.

CAC was classified as being minor, mild, moderate, or severe, according to the Agatston scores. A score less than 10 was classified as being minor CAC, a score between 10 and 100 was classified as being mild CAC, a score between 100 and 400 was classified as being moderate CAC, and a score of more than 400 was classified as being severe CAC.

### Statistical analysis

Data were analyzed by using SPSS software version 17 (SPSS Inc., Chicago, IL). The data are presented as the mean ± SD for the normally distributed variables; otherwise, the data are presented as the median and 25th–75th percentiles. Differences between the two groups were compared by using either independent *t* tests or Wilcoxon’s rank tests for the continuous variables. Frequencies between the two groups were analyzed by using the chi-square test. A binary logistic regression model was used to screen the variables for CAC (no calcification: a calcification score of 0; calcification: a CAC score of 1 or more), and the variables that were correlated *via* the univariate regression analysis or that were known to be important in the physiology of vascular calcification were selected (*p* ≤ .1). In our study, a two-tailed *p* value ≤ .05 was set to indicate a statistically significant difference.

## Results

Two hundred ninety CKD patients were enrolled in our study; 168 patients with CAC and 122 patients without CAC. Among them, 139 were non-dialysis CKD patients and 151 were dialysis patients. There were 141 hemodialysis patients and 10 peritoneal dialysis patients. There were 73 cases in the mild CAC group, 43 cases in the moderate CAC group, and 52 cases in the severe CAC group. Additionally, there were 45 healthy controls in the control group. No significant differences were observed in age, or male proportion between the two groups (*p >* .05). Blood samples from only 131 CKD patients were collected, and the parameters of IL-6 and MGP were measured. There was higher IL-6 level in case group than that in healthy control group (*p* < .001), lower MGP level was observed in case group (*p* = .027). These data are shown in Table 1.

**Table 1.** The comparisons of clinical parameters between CKD patients and control group.

Variables	Case group (*n* = 290)	Control group (*n* = 45)	*t*/z/*x*^2^	*p* Value
Male [*n* (%)]	173 (59.66%)	24 (54.33%)	0.643	.515
Age (years)	54 (47, 64）	55 (49.50, 66.5）	−0.916	.360
Hb (g/l)	104.66 ± 24.96	141.13 ± 15.308	−9.53	<.001
IL-6 (pg/ml)	32.02 (12.89, 91.20)	3.85 (2.33, 5.42)	−8.167	<.001
MGP (ng/ml)	11.69 (8.53, 16.79)	15.78 (9.65, 17.03)	−2.216	.027

CKD: chronic kidney disease; Hb: hemoglobin; IL-6: interleukin 6; MGP: matrix Gla protein

There was 131 CKD patients measured for IL-6 and MGP levels.

The parameters of IL-6 and MGP were evaluated in the non-CAC group (*n* = 35), the mild CAC group (*n* = 48), the moderate CAC group (*n* = 25), and the severe CAC group (*n* = 23). The levels of IL-6 in the severe CAC group were significantly higher than that in the mild to moderate CAC group (*p* < .001), MGP levels were lower in severe CAC group than that in the mild to moderate CAC group (*p* < .001).

The levels of IL-6 and MGP were measured in non-CAC group (*n* = 35), in CAC group (*n* = 96). The level of serum IL-6 was higher in the case group than that in the non-CAC group [48.18 (20.28, 118.05) pg/ml *versus* 10.51 (5.65, 23.46) pg/ml, *p* < .001]. A lower MGP concentration was observed in the CAC group than in the non-CAC group [10.50 (8.49, 13.53) ng/ml *versus* 18.88 (13.41, 23.23) ng/ml, *p* < .001]. As seen in Table 2.

**Table 2.** The comparison of clinical characters between CAC and non-CAC groups.

Variables	CAC group (*n* = 168)	Non-CAC group (*n* = 122)	*t*/*z*/*x*^2^	*p* Value
Age (years)	55 (48.25, 64.75)	52 (44, 57.75)	−3.263	.001
Male (*n*)	99	74	0.148	.714
Hypertension (*n*)	127	80	3.474	.067
Diabetes mellitus (*n*)	57	19	11.799	<.001
Smoking (*n*)	28	14	0.823	.364
RRT (*n*)	104	47	15.48	<.001
Phosphate binders (*n*)	92	71	0.339	.561
Vitamin D analog or calcimimetics	81	68	1.601	.206
Hb (g/l)	105.49 ± 25.50	103.44 ± 23.89	0.678	.499
NLR	4.02 (2.79, 5.61)	2.8 (1.97, 3.71)	–5.918	<.001
Scr (µmol/l)	752.94 ± 365.927	669.38 ± 339.48	–1.919	.088
UA (µmol/l)	363.56 ± 116.25	363.16 ± 104.537	–0.018	.986
Ca (mmol/l)	2.14 ± 0.27	2.11 ± 0.29	–0.707	.480
P (mmol/l)	1.97 ± 0.59	1.63 ± 0.49	–4.805	<.001
Mg (mmol/l)	0.98 (0.85, 1.10)	0.96 (0.85, 1.09）	–0.986	.324
iPTH (pg/ml)	668 (250, 1486)	237.5 (143, 487.25）	–5.458	<.001
hs-CRP (mg/l)	4.7 (2.30, 10.08)	3.45 (1.57, 6.89)	–2.260	.024
25 (OH)D3 (ng/ml)	18.6 (12.7, 25.03)	17.12(10.13, 21.58)	–1.971	.049
ALP (U/l)	125 (89.25, 234.75)	98 (68, 150)	–3.992	.001
ALB (g/l)	34.8 (29.53, 38.08)	36.4 (33.4, 39.1)	–2.549	.011
TC (mmol/l)	3.74 (3.24, 4.73)	4.21 (3.46, 5.23)	–2.242	.025
TG (mmol/l)	1.2 (0.93, 2.08)	1.28 (0.96, 2.03)	–0.393	.694
Dialysis duration (month)	29 (12, 72)	11 (7, 24)	–4.288	<.001
IL-6 (pg/ml)	48.18 (20.28, 118.05)	10.51 (5.65, 23.46)	–5.165	<.001
Chemerin (ng/ml)	91.05 (61.51, 113.31)	93.45 (82.77, 112.93)	–0.906	.365
MGP (ng/ml)	10.50 (8.49, 13.53)	18.88 (13.41, 23.23)	7.516	<.001

RRT:renal replacement therapy; Hb: hemoglobin; NLR: neutrophil to lymphocyte ratio; Scr: serum creatinine; Ca: calcium; P: phosphorus; Mg: magnesium; iPTH: intact parathyroid hormone; hs-CRP: high-sensitivity C-reactive protein; TC: total cholesterol; TG: triglyceride

The levels of IL-6 and MGP were measured in non-CAC group (*n* = 35), in CAC group (*n* = 96).

The total prevalence of CAC was 57.93%, and the prevalence in dialysis patients (up to 68.87%) was higher than that in non-dialysis patients. Patients were older in the CAC group than that in the non-CAC group (*p* < .001). Furthermore, the proportion of diabetes patients in the CAC group was significantly higher than that in the non-CAC group. Additionally, the value of NLR was significantly higher in the CAC group than that in the non-CAC group (*p* < .001). The CAC group also had higher levels of phosphorus and iPTH than the non-CAC group. There were 71 patients taking phosphate binders in non-CAC group, 92 patients taking phosphate binders in CAC group (*p* = .561). There were 68 patients receiving vitamin D analog or calcimimetics in non-CAC group, 81 patients receiving vitamin D analog/calcimimetics in CAC patients (*p* = .206). As seen in Table 2.

To explore the factors for CAC, we conducted a binary logistic regression analysis. In the univariate logistic analysis, variables with a *p* value less than .1, such as age, hypertension, diabetes mellitus, serum phosphorus, iPTH, 25(OH)D3, ALP, NLR, Alb, and CKD stage (dialysis or not) were included in the multivariate logistic regression analysis (as shown in Table 3).

**Table 3.** Univariate logistic regression analysis of risks for CAC in patients with CKD.

				OR (95% CI)	
Variables	*β*	*p*	OR	Lower	Upper
Sex	0.072	.767	1.074	0.668	1.729
Age	0.764	.002	2.146	1.326	3.474
Hypertension	0.486	.063	1.626	0.973	2.717
Diabetes mellitus	1.004	.001	2.730	1.521	4.900
Smoking	0.381	.280	1.464	0.733	2.921
Hb (g/l)	0.003	.509	1.003	0.994	1.013
NLR < 2.67		.000			
NLR 2.67–4.22	1.475	.000	4.370	2.415	7.906
NLR > 4.22	2.245	.000	9.44	4.740	18.815
hs-CRP (mg/l)	0.001	.870	1.001	0.989	1.013
Ca (mmol/l)	0.316	.469	1.372	0.583	3.229
P (mmol/l)	1.006	.000	2.902	1.808	4.659
iPTH (pg/ml)	0.001	.000	1.001	1.001	1.002
Mg (mmol/l)	0.042	.358	1.043	0.954	1.140
25(OH)D_3_ (ng/ml)	0.022	.054	1.022	1.000	1.045
ALP (U/l)	0.002	.007	1.002	1.000	1.003
Alb (g/l)	−0.039	.045	0.962	0.926	0.999
TC (mmol/l)	−0.215	.022	0.806	0.670	0.970
TG (mmol/l)	0.120	.159	1.128	0.954	1.333
CKD stage	0.953	.000	2.593	1.605	4.188
Dialysis duration (month)	0.020	.000	1.021	1.011	1.031
IL-6 (pg/ml)	0.021	.001	1.022	1.008	1.035
MGP (ng/ml)	−0.254	.000	0.775	0.705	0.853

Variable assignment: age (age < 50 years old = 1, age ≥ 50 years old = 2); NLR (NLR < 2.67 = 1, NLR 2.67–4.22 = 2, NLR >4.22 = 3).

The levels of IL-6 and MGP were measured in non-CAC group (*n* = 35), in CAC group (*n* = 96).

First, a multivariate logistic regression analysis was conducted in 290 patients, and older age was associated with an increased risk for CAC (OR = 2.701, *p* = .004). Diabetes mellitus, hyperphosphatemia, dialysis duration, NLR were positively correlated with an increased risk for CAC. Moreover, we classified the NLR value into high, moderate, low level according to tertiles. Compared with low value of NLR (<2.62), high level group (NLR > 4.22) had 10-fold higher risk for CAC. By binary logistic regression, age, diabetes mellitus, hyperphosphatemia, dialysis duration, and NLR were positively associated with CAC. Second, a binary logistic regression was performed on 131 patients. In addition to the previously described risks being related to CAC, higher serum IL-6 levels were positively associated with CAC (OR = 1.015, *p* = .038), and lower serum MGP levels were correlated with CAC risk (OR = 0.766, *p* < .001). These results are shown in Table 4.

**Table 4.** Multivariate logistic regression analysis of risks for CAC in patients with CKD.

				OR (95% CI)	
Variables	*β*	*p*	OR	Lower	Upper
Age	0.993	.004	2.701	1.368	5.331
Diabetes mellitus	1.046	.009	2.846	1.292	6.270
P (mmol/l)	1.094	.001	2.985	1.561	5.707
Dialysis duration	0.014	.009	1.014	1.003	1.025
NLR <2.67		.000			–
NLR 2.67– 4.22	1.447	.013	4.252	2.086	8.668
NLR > 4.22	2.471	.001	11.83	4.960	28.213
IL-6 (pg/ml)	0.015	.038	1.015	1.001	1.030
MGP (ng/ml)	–0.267	.000	0.766	0.660	0.889

Variable assignment: age (age < 50 years old = 1, age ≥ 50 years old = 2; NLR (NLR < 2.67 = 1, NLR 2.67–4.22 = 2, NLR > 4.22 = 3).

**Table 5.** Multivariate cox regression analysis of risks for CVE in patients with CKD.

				HR (95% CI)	
Variables	*β*	*p* Value	HR	Lower	Upper
Moderate to severe CAC	1.981	.001	7.250	3.192	16.470
P (mmol/l)	1.162	.001	3.198	2.026	5.049
MGP (ng/ml)	−1.080	.036	0.340	0.124	0.933

The follow-up visits were 3–19 months intervals (median 11 months). A total of 290 patients with CKD were followed up by telephone, and 14 patients were lost to the follow-up, with a total of 48 cardiovascular events. There were 23 cases of heart failure, six cases of myocardial infarction, four cases of malignant arrhythmia, four cases of new cerebral hemorrhage, and eight cases of new cerebral infarction. Two patients received interventional therapy due to lower limb artery stenosis, and one patient underwent amputation because of a serious infection.

**Figure 1.**
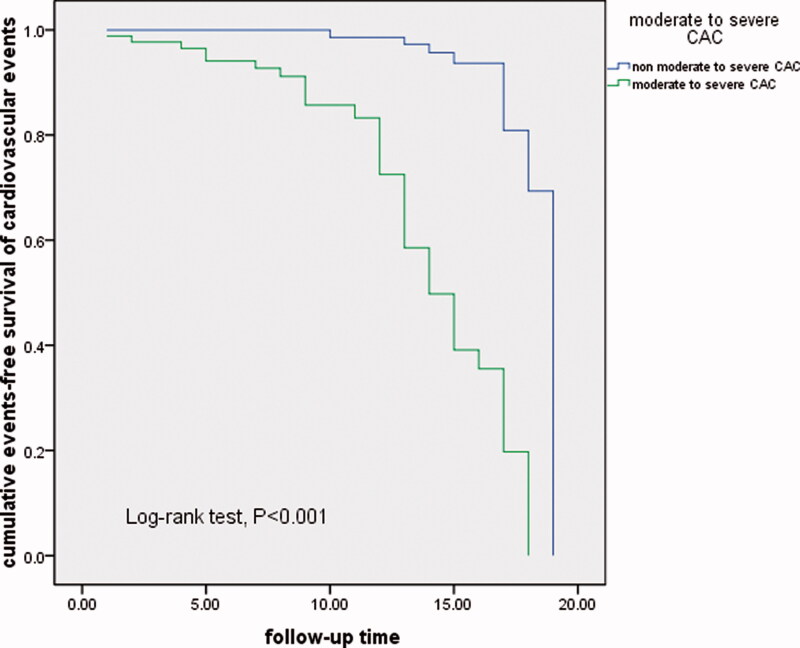
Kaplan–Meier curve for non-fatal cardiovascular events according to coronary artery calcification score; group with moderate to severe CAC (CACS ≥ 100), group with non-moderate to severe group (CACS<100).

**Figure 2.**
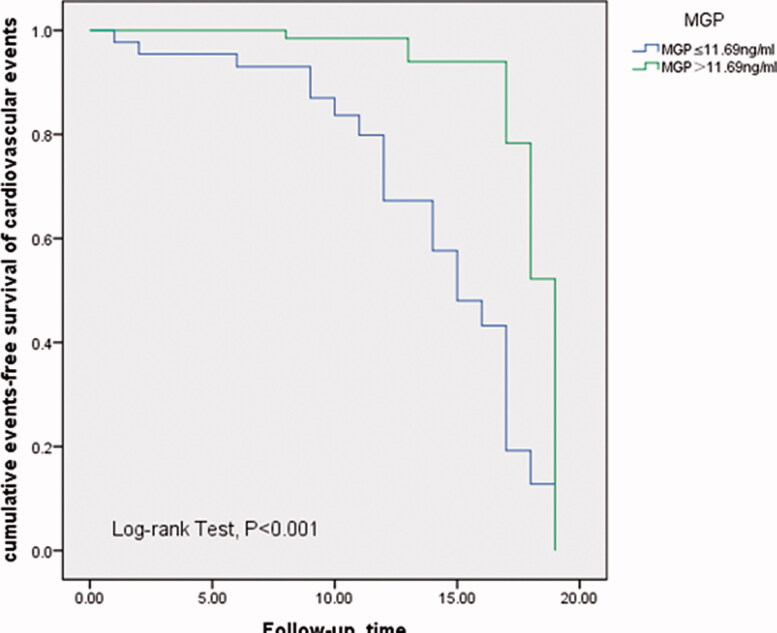
Kaplan–Meier curve for non-fatal cardiovascular events according to MGP levels; group with above-median MGP (MGP > 11.69 ng/ml), group with below-median MGP (MGP ≤ 11.69 ng/ml).

To evaluate the risk factors for cardiovascular events in patients with CKD, a univariate cox analysis was performed. The *p* values less than .1 in the univariate Cox analysis were included in the multivariate Cox regression analysis. In the multivariate Cox regression analysis for cardiovascular events in 276 CKD patients, moderate to severe CAC was associated with an increased risk for CVE (HR:7.250; 95% CI: 3.192–16.470), hyperphosphatemia was related to an increased risk for CVE (HR: 3.198; 95% CI: 2.026–5.049). Patients tested for both parameters were followed up. In addition to the above-described risks being correlated with CVE, a higher MGP level was inversely associated with a reduced risk for CVE (HR: 0.340, 95% CI: 0.124–0.933). These results are shown in Table 5. Kaplan–Meier analysis demonstrated that moderate to severe CAC contributed to CVE (log-rank test, *p* < .001), as shown in Figure 1. Kaplan–Meier analysis revealed that lower MGP level was related to an increased risk for CVE (log-rank test, *p* < .001) as shown in Figure 2.

## Discussion

In our study, it was demonstrated that CAC was common in CKD patients; moreover, older age, hyperphosphatemia, dialysis duration, diabetes mellitus, IL-6, and NLR were associated with the prevalence of CAC. Higher MGP level was a protective factor for CAC. We found that moderate to severe vascular calcification resulted in cardiovascular events. Therefore, we should take measures to prevent the progression of CAC and to reduce cardiovascular events, in order to improve the prognosis of CKD patients. The mechanism for CAC progression is not fully understood; therefore, it is very important for us to explore the risk factors.

The incidence of diabetes in China has been increasing in recent years. Patients with diabetes mellitus have a low control rate of blood glucose. In our logistic analysis, a history of diabetes mellitus was positively associated with CAC. The mechanism of cardiovascular calcification caused by diabetes mellitus can be related to several different factors. For example, advanced glycosylation end products (AGEs) are the main metabolites of diabetes patients, which can increase the oxidative stress response and contribute to the vascular smooth cell (VSMC)-to-osteoblast-like transition [12]. Additionally, the extracellular regulated protein kinase (ERK1/2) and nuclear factor-kappa gene binding (NF-κB) pathways can become activated, and Runt-related transcription factor 2 (Runx2) expression can also become upregulated [13].

Cardiac valvular calcification was shown to be positively associated with age. In an animal model, the artery calcification was more severe at 48–72 weeks than that at 24 weeks [14]. Under uremic conditions, VSMCs tend to accelerate aging, and aging VSMCs tend to transdifferentiate toward osteoblast-like cells. We found that older age obviously increased the risk for CAC.

By binary logistic analysis, we found that the levels of IL-6 were significantly associated with CAC. IL-6 promotes the expression of Runx2, which induces VSMCs to develop osteoblast-like characteristics [15].

By binary logistic analysis, we demonstrated that NLR was significantly correlated with an increased risk for CAC. The mechanism by which the NLR results in an increased risk for CAC involves the fact that neutrophils can release a variety of inflammatory mediators and cytokines, thus leading to chronic microinflammation and deteriorating vascular calcification [16]. Moreover, neutrophils activate peroxidase and lead to increased production of oxygen free radicals. Second, the expression of matrix metalloproteinases becomes upregulated in these circumstances, which results in the degradation of extracellular matrix components, such as collagen fibers and elastic fibers. The degradation products can then accelerate vascular smooth muscle transdifferentiation into osteoblastic cells, thus contributing to vascular calcification. Third, lymphocyte apoptosis mediates the production of inflammatory mediators and accelerates vascular calcification [17].

Luo G. et al. found that MGP gene knockout mice can develop artery calcification after two months and eventually die because of ruptured blood vessels and heart failure [10]. MGP has also been shown to inhibit the formation of apoptotic bodies, and cell experiments have confirmed that apoptotic bodies can initiate vascular calcification [18,19]. The formation of matrix vesicles was prevented by MGP [20]. Matrix vesicles can bind to intracellular and extracellular calcium ions, thus reducing the formation of stromal vesicles, and ultimately inhibiting the development of vascular calcification. Schlieper G. et al. found that low carboxylated MGP levels can contribute to all-cause mortality and cardiovascular mortality [21]. In our study, lower MGP levels were observed in CAC group, and increased the risk for cardiovascular events. There was other interesting papers analyzing the relationship between biomarkers and cardiovascular events, demonstrated that lower adiponectin predicted cardiovascular events in dialysis patients [22].

Due to the short follow-up time and few observed deaths, a correlation analysis was not conducted between serum MGP levels and death. However, we found that low MGP levels were associated with CAC. In our follow-up, we found that moderate to severe CAC increased the risk for cardiovascular events, and the risk increased approximately seven times compared with the non-CAC group. It has been speculated that CAC may occur in the intimal and medial membrane of the artery. Severe coronary atherosclerosis may lead to myocardial ischemia or even myocardial infarction in patients with CKD. Additionally, calcification in the artery medial membrane may result in decreased vascular compliance, left ventricular hypertrophy, or heart failure in severe cases.

The calcification burden of CKD is affected by both traditional (i.e., older age, diabetes, and smoking habits), and nontraditional (i.e., inflammation, oxidative stress, and disordered mineral metabolism) cardiovascular risk factors. In this study, diabetes, older age, and high phosphorus levels were associated with CAC. We could not demonstrate a correlation between certain risk factors and CAC, such as hypertension and higher calcium levels. This may be due to the small sample size and cross-sectional nature of our study, as well as lower PTH levels caused by vitamin D and analog.

There were some additional limitations in our study. First, this study demonstrated an association between elevated IL-6/NLR and CAC, a definitive causal relationship was not established. Second, all of the patients were in the hospital, and they required CAC examination; therefore, there may be some selective deviation in our study. Third, we could not identify a relationship between certain risks and death because of the short follow-up visits.

In conclusion, higher prevalence of CAC in CKD patients is found, and is affected by several factors. Moderate to severe CAC predicts higher incidence of cardiovascular events in the short follow-up intervals.
